# The Effect of rGO-Doping on the Performance of SnO_2_/rGO Flexible Humidity Sensor

**DOI:** 10.3390/nano11123368

**Published:** 2021-12-12

**Authors:** Huangping Yan, Zilu Chen, Linyuan Zeng, Zijun Wang, Gaofeng Zheng, Rui Zhou

**Affiliations:** 1 School of Aerospace Engineering, Xiamen University, Xiamen 361005, China; chenzilu@szpt.edu.cn (Z.C.); 34520182201727@stu.xmu.edu.cn (L.Z.); 35120191151196@stu.xmu.edu.cn (Z.W.); zheng_gf@xmu.edu.cn (G.Z.); rzhou2@xmu.edu.cn (R.Z.); 2 Shenzhen Research Institute of Xiamen University, Shenzhen 518000, China

**Keywords:** SnO_2_/rGO nanocomposite, humidity sensor, flexibility, breath monitoring

## Abstract

The development of a flexible and high-performance humidity sensor is essential to expand its new applications, such as personal health monitoring and early diagnosis. In this work, SnO_2_/rGO nanocomposites were prepared by one-step hydrothermal method. The effect of rGO-doping on humidity sensing performance was investigated. Scanning electron microscopy, transmission electron microscopy, X-ray diffraction and Raman spectroscopy were used to characterize the nanostructure, morphology and chemical composition of SnO_2_/rGO nanocomposites. The SnO_2_/rGO humidity sensitive film was prepared by electrospinning on a polyimide film modified with gold electrodes. The humidity test results show that different doping ratios of rGO have different effects on humidity sensing properties. Among them, the sensor with 2 wt% rGO-doping has a high sensitivity (37,491.2%) within the humidity range as well as the fast response time (80 s) and recover time (4 s). Furthermore, the sensor with 2 wt% rGO-doping remains good flexibility and stability in the case of bending (1000 times). The sensitivity of the 2 wt% rGO-doping sensor at the bending radius (8 mm and 4 mm) is 48,219% and 91,898%, respectively. More importantly, the sensor could reflect different breathing states clearly and track breathing intervals as short as 3 s. The SnO_2_/rGO flexible humidity sensor with accuracy, flexibility and instantaneity as well as the facile fabrication strategy is conceivable to be applied in the potential application for human health real-time monitoring.

## 1. Introduction

Nowadays, accurate detection of humidity is important for industrial production and home life [1,2,3]. With people’s increasing attention to personal health, the application fields of humidity sensors are expanding rapidly, including real-time monitoring of human breathing rate [4,5], sweat management [6], noncontact switch [7], etc. [8,9]. The prerequisite for meeting the above-mentioned applications is excellent humidity sensing characteristics. Thus, the synthesis of sensitive materials is still the key to increase the performance of humidity sensors. Over the years, researchers have explored many new high-performance sensitive materials in terms of material types and structures [10,11,12,13].

Among the metal oxides, SnO_2_ is a n-type semiconductor material with a wide band gap, which is considered as a promising material for humidity monitoring due to its intrinsic chemical property and low cost [14,15]. In order to obtain good sensing properties, SnO_2_ in different nanostructures have been studied, such as mesoporous SnO_2_ nanostructures and SnO_2_ nanowires [16,17]. Nevertheless, the low sensitivity of pure SnO_2_ humidity sensor under high relative humidity limits its practical application [18]. Graphene, as a 2D nanosheet, has the remarkable chemical and physical properties, which are widely used in a variety of fields [19,20]. The large specific surface area and efficient electrons transfer of graphene and its derivatives (GO and rGO) strongly promote the reaction process with water molecules, but they are not conductive to real-time humidity detection because of the long response and recover time. In recent years, some investigations have demonstrated that combining SnO_2_ with graphene or its derivatives to prepare nanocomposites for humidity sensing could increase the direct contact region between water molecules and nanostructures and reduce the energy barrier, which effectively improves the humidity sensing performance [21,22,23,24]. Xu et al. prepared SnO_2_@G-GO composites using electrospinning method and solution evaporation method in succession. Compared with pure SnO_2_, the SnO_2_@G-GO was demonstrated better humidity characteristics with high sensitivity, fast response and good stability [22]. D. Toloman et al. demonstrated that the prepared SnO_2_: Fe-graphene composites humidity sensor showed a higher sensitivity than Fe: SnO_2_ nanoparticles [23]. Most of the literature has focused on the facile preparation method and new doping materials. However, the effects of different doping ratios on sensing properties of nanocomposites have not been further studied.

Traditional humidity sensors are usually prepared on rigid substrates [25,26,27] with some shortcomings such as easy cracking of the electrode, poor stability and incompatibility with human skin, which limit the scope of application. A variety of flexible substrate materials (e.g., polyethylene terephthalate [28], polyimide [29,30] and paper [31]) with the advantages of low cost, favorable mechanical flexibility and good biocompatibility have attracted more and more attention. Researchers have taken kinds of methods to fabricate flexible humidity sensors in order to improve the compatibility of flexible sensors with skin surface and even to better adapt to a variety of environmental stimuli, including temperature, pressure, and lateral strain [32,33,34,35]. Shinya Kano et al. reported that a flexible humidity sensor fabricated by spin-coating the all-inorganic colloidal silicon nanocrystals. Due to the fast response/recovery time, the sensor could be used in real-time monitoring of human’s breath and skin moisture [36]. Zhang et al. deposited MoS_2_-SnO_2_ nanocomposites on the polyimide (PI) film by a drop cast method to prepare an ultrasensitive humidity sensor. The sensor revealed an unprecedented response up to 3,285,000% at 100 Hz, ultrafast response/recovery behaviors and outstanding repeatability [37]. However, in order to maximize the good performance of sensitive materials, the process of preparing humidity-sensitive film usually requires complicated operation and high costs. Adopting a facile and low-cost method to transfer sensitive materials to a flexible substrate while improving good sensing performance is one of the urgent issues to be addressed.

Here, SnO_2_/rGO nanocomposites with different doping ratios of rGO were synthesized by a one-step hydrothermal method as sensing materials. The sensitive film of SnO_2_/rGO was formed on the PI film by an electrospinning method to prepare the humidity sensor. The influence of different rGO-doping ratios on the humidity sensing properties were studied in a range of 11%RH–95%RH at room temperature (20 °C). The sensing mechanism of SnO_2_/rGO nanocomposite was explored. The prepared sensor shows good performance including high sensitivity, good flexibility and repeatability. In addition, the flexible sensor represents good performance in human respiration monitoring. The prepared humidity sensor has great potential in medical care, wearable devices and other intelligent fields.

## 2. Materials and Methods

### 2.1. Preparation of SnO_2_/rGO

SnO_2_/rGO nanocomposites were synthesized by a simple one-step hydrothermal method. The GO aqueous dispersion (1 mg/mL, Xilong Scientific Co. Ltd., Foshan, Guangdong, China) and 0.1 g SnCl_4_·5H_2_O (>98 wt%, Xilong Scientific Co. Ltd., Foshan, Guangdong, China) were added into 20 mL deionized water. The solution was dispersed by an ultrasonic method for 30 min. The sodium citrate solution (Xilong Scientific Co. Ltd., Foshan, Guangdong, China) was added to adjust the pH value of the mixed solution. These chemicals are analytically pure in specification. Then, the mixed solution was transferred to an autoclave lined with Teflon and heated in an electric furnace at 180 °C for 12 h. The sample was then centrifuged and washed several times, and finally preserved in the form of a disperse solution. SnO_2_/rGO with different rGO-doping ratios (1 wt%, 2 wt%, 4 wt%, 8 wt%) were prepared by the same method.

### 2.2. Fabrication of a Flexible Humidity Sensor

**Figure 1:**
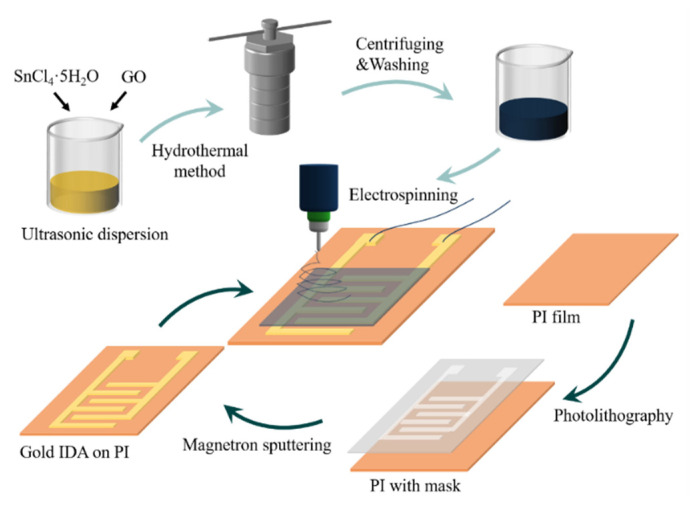
Schematic diagram of SnO_2_/rGO humidity sensor preparation process.

First, PI sheets (12 mm × 8 mm × 75 μm) were successively cleaned by ultrasonic in acetone, ethanol and deionized water for about 5–10 min. Then, the sheets were blown dry by nitrogen (N_2_). The Au (30 nm Cr thin layer before 300 nm Au layer sputtering) interdigitated electrode arrays (IDA) were fabricated by magnetron sputtering (Explorer-14, Denton Vacuum, Denton, TX, USA) combined with MEMS technology on flexible PI sheets. The number of electrode pairs was six. The width of every single electrode is 100 μm and the interfinger gap between the electrodes was 150 μm. Before deposition, the synthesized SnO_2_/rGO nanocomposites was sonicated to enable it to be properly dispersed on the IDA. Further, the samples were sprayed on PI sheets using electrospinning method (AC 10 V, 100 Hz, square wave, serpentine spray paths, spacing 0.5 mm, repeat 50 times). Lastly, the prepared sensor was dried in electrothermal blowing dry box at 60 °C for 1 h. Figure 1 is the schematic diagram of the preparation process of SnO_2_/rGO humidity sensor.

### 2.3. Characterization and Humidity Performance Test

The microstructure and morphology of SnO_2_/rGO nanocomposites were imaged by a field emission scanning electron microscopy (SEM, ZEISS SUPRA 55, Carl Zeiss, Oberkochen, Germany) and a field emission transmission electron microscopy (TEM, Jem-2100, JEOL, Tokyo, Japan). The Raman spectra were obtained by Raman spectrometer (Raman, iDSpec ARCTlC, iDSpec, Beijing, China). The structure and crystallite size were analyzed by a powder X-ray diffraction (XRD, XRD-7000, Shimadzu, Kyoto, Japan) within the 2θ range of 20° to 80°. In terms of humidity test, specific RH conditions of 11%, 20%, 34%, 56%, 77%, and 95% were obtained in wide mouthed bottles with saturated solutions containing a variety of metal salts including LiCl, CH_3_COOK, MgCl_2_, NaBr, KCl, and CuSO_4_·5H_2_O. The capacitance of SnO_2_/rGO humidity sensor under different RH levels was measured with an impedance analyzer (LCR, AT3818, Anbai, Changzhou, China) at AC 0.5 V and 100 Hz. All the properties tests were carried out at 20 °C. The humidity sensor sensitivity is defined as S = (C_x_ − C_0_)/C_0_ × 100%, where C_x_ and C_0_ represent the capacitance at the specific humidity X%RH and initial humidity 11%RH, respectively. The time required for the capacitance value of the sensor to reach 90% of the final equilibrium value during the absorption and desorption process is defined as the response and recovery time, respectively.

## 3. Results

### 3.1. Material Characterizations

**Figure 2:**
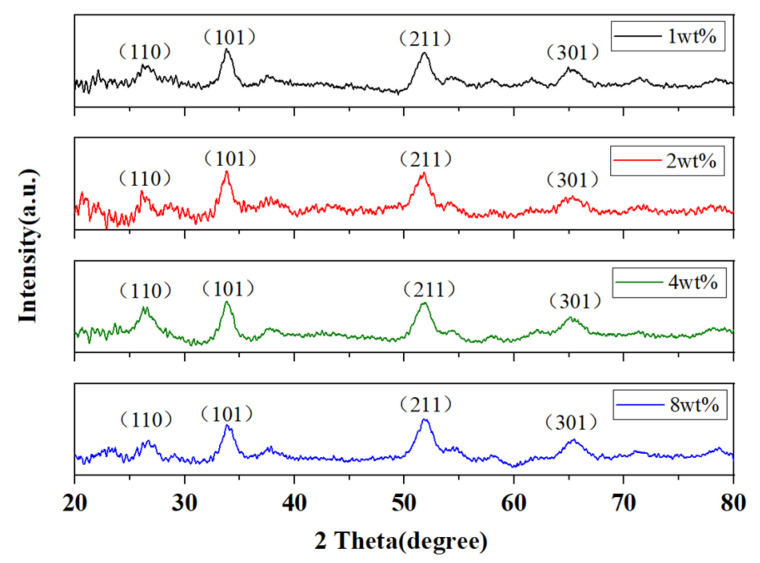
XRD of SnO_2_/rGO nanocomposites samples.

XRD patterns, as shown in Figure 2, were recorded to identify the crystal structure of synthesized samples. The diffraction peaks of SnO_2_ are corresponding to the (110), (101), (211) and (301) diffraction of tetragonal cassiterite structure SnO_2_ (JCPDS 41-1445) [38], which illustrates SnO_2_ was formed. The diffraction peak of rGO is about 25°, which is close to 26° and coincides with the diffraction peak of (110) crystal plane of SnO_2_, so additional characterization is required for further analysis of rGO.

**Figure 3:**
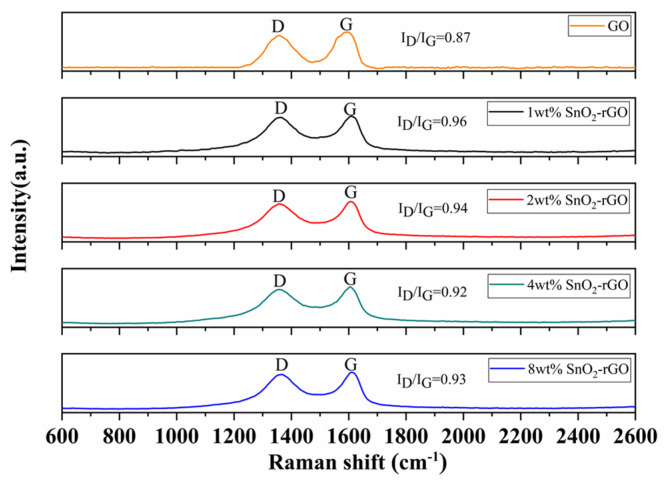
Raman spectra of GO and SnO_2_/rGO nanocomposites samples.

The Raman spectra were displayed in Figure 3. In general, carbon materials have two characteristic broad peaks named D band and G band near 1356 cm^−1^ and 1580 cm^−1^. The D band is attributed to the defects and disordered structures in graphene, and the G band is derived from the in-plane vibration of carbon atoms in the sp^2^ -hybrid orbital. The intensity ratio of D band to G band (I_D_/I_G_) stands for the density of defects for the graphene structure [38]. As shown in Figure 3, the I_D_/I_G_ value of 1 wt% SnO_2_-rGO is 0.96 which is greater than that of GO (0.87), indicating that GO was reduced to rGO upon the hydrothermal method and the defect structures of the composites increased. The structural defects could provide more active sites for adsorption and desorption of water molecules. In addition, it can be seen that the I_D_/I_G_ value of 1 wt% SnO_2_-rGO is the largest and that of 4 wt% SnO_2_-rGO is the smallest, which also corresponds to the subsequent humidity test results.

The position of G band for SnO_2_/rGO composites reveals the interaction between rGO and SnO_2_. From the Raman spectra, the G band shift of the SnO_2_/rGO composites indicated the formation of SnO_2_/rGO composites rather than individual presence during the process of the hydrothermal synthesis, which is attributed to the charge transfer between rGO and SnO_2_ [39].

**Figure 4:**
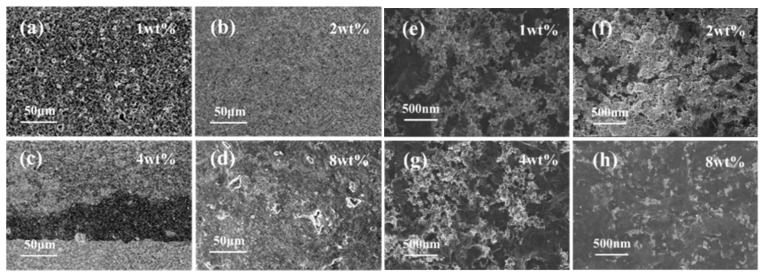
SEM images of SnO_2_/rGO nanocomposites. SEM images of (**a**) 1 wt% SnO_2_/rGO, (**b**) 2 wt% SnO_2_/rGO, (**c**) 4 wt% SnO_2_/rGO, and (**d**) 8 wt% SnO_2_/rGO at low magnification; SEM images of (**e**) 1 wt% SnO_2_/rGO, (**f**) 2 wt% SnO_2_/rGO, (**g**) 4 wt% SnO_2_/rGO, and (**h**) 8 wt% SnO_2_/rGO at high magnification.

Figure 4a–d shows SEM images of SnO_2_/rGO nanocomposites at low magnification. There are some differences in microscopic morphology of samples with different doping ratios. Compared with other samples, the 2 wt% SnO_2_/rGO film prepared by electrospinning is more uniform, while the 4 wt% SnO_2_/rGO film is most affected by the electric field and therefore has the worst uniformity. Figure 4e–h shows SEM images of SnO_2_/rGO nanocomposites at higher magnification. For 1 wt% and 2 wt% SnO_2_/rGO, SnO_2_ nanoparticles are stacked on graphene to form a porous and loose structure, which is more beneficial for effective surface reactions. As the proportion of graphene doping increases, most of the nanoparticles are sparsely dispersed on the multi-layer structure of graphene, especially the micromorphology of 8 wt% SnO_2_/rGO.

**Figure 5:**
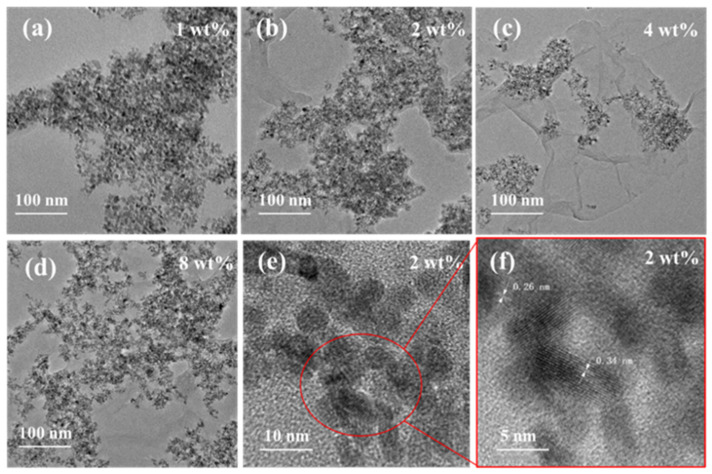
TEM images of SnO_2_/rGO nanocomposites samples. (**a**) 1 wt% SnO_2_/rGO, (**b**) 2 wt% SnO_2_/rGO, (**c**) 4 wt% SnO_2_/rGO, (**d**) 8 wt% SnO_2_/rGO, (**e**,**f**) HRTEM images of 2 wt% SnO_2_/rGO nanocomposites.

Structural analysis with more details of SnO_2_/rGO was achieved by using TEM and HRTEM as shown in Figure 5. At low magnification, TEM images in Figure 5a–d reveal the SnO_2_ nanoparticles were scattered on the rGO nanosheets. Among the four samples with different doping ratios, the distribution of nanoparticles of 4 wt% SnO_2_/rGO is the most uneven, which is consistent with the results of SEM images in Figure 4c. The HRTEM image in Figure 5e shows that the diameter of SnO_2_ nanoparticles were around 8 nm. Furthermore, it could be calculated that the d spacings of the nanoparticles is 0.34 nm and 0.26 nm from Figure 5f, which is in accord with the d spacings of (110) and (101) of SnO_2_ [38].

### 3.2. Humidity Sensing Properties

The method of saturated salt solution is used to build the different humidity environments [40]. The humidity sensing testing setup is mainly composed of humidity source bottles, a LCR analyzer and a computer. The sensor is placed into the different humidity bottles to sense the different humidity. The capacitance of SnO_2_/rGO humidity sensor under different RH levels is measured by a LCR analyzer. The parallel capacitance equivalent circuit of digital bridge is used to measure the capacitance by constant voltage mode.

The influence of operating frequency on humidity response characteristics were investigated. The sensor was transferred from low humidity environment to high humidity environment (11%–20%–34%–56%–77%–95%) successively and the capacitance response values were measured at 100 Hz, 1 KHz, 10 KHz, 100 KHz and 200 KHz, respectively. The capacitance decreased with increasing frequency. The results showed that the capacitance change of the sensor at 100 Hz is significantly better than that at other working frequencies. Especially, there is a sudden increase of the capacitance in high humidity. Therefore, 100 Hz is chosen as the operating frequency of performance test.

**Figure 6:**
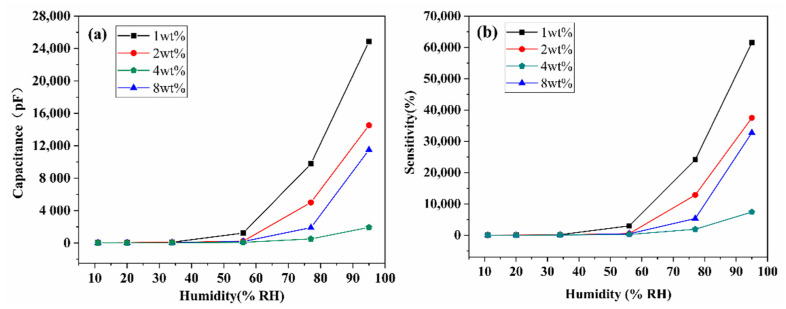
The influence of different doping ratios on (**a**) capacitance and (**b**) sensitivity of SnO_2_/rGO sensor in the range of 11%RH–95%RH.

The capacitance value curves of four samples with relative humidity at 20 °C are shown in Figure 6a. At 100 Hz, the capacitance increases monotonically with the rise of RH, indicating that the capacitance response is promoted with the increase of water molecule adsorption. Among them, the capacitance of the 1 wt% SnO_2_/rGO sensor is found to be about 24,866 pF at 95%RH, which is about 616 times of that (40.36 pF) at 11%RH. For the purpose of comparing the humidity sensing property of four samples, the sensitivity is calculated as shown in Figure 6b. It is obvious that the sensitivity of all kinds of SnO_2_/rGO humidity sensors increases sharply by more than three orders of magnitude from 11%RH to 95%RH, indicating that water molecules have a great effect on the capacitance of SnO_2_/rGO nanocomposites. Especially, 1 wt% SnO_2_/rGO sensor corresponds to a higher sensitivity (61,510%) to water molecules than other samples due to the honeycomb-like structure on the film. The honeycomb-like structure with large pore size increases the specific surface area of the sample, which provide more available active sites for the adsorption of water molecules.

**Figure 7:**
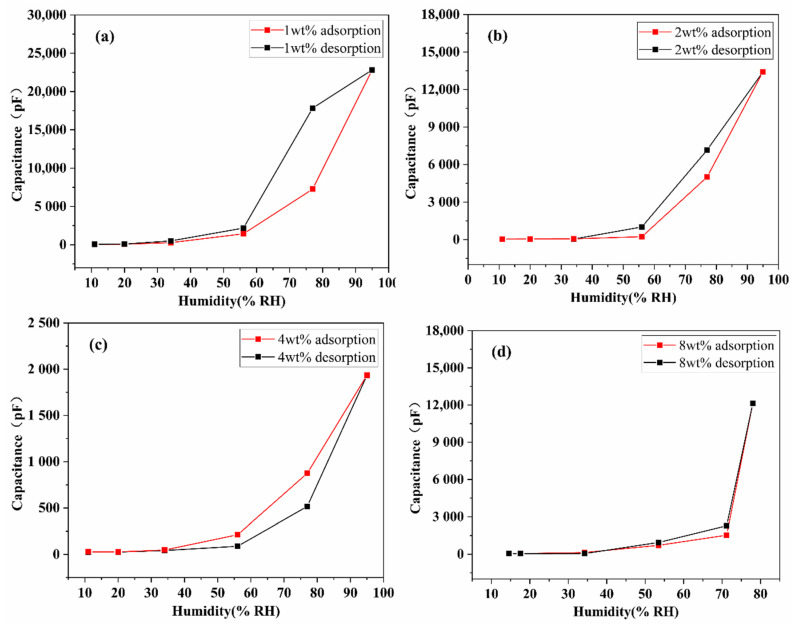
Hysteresis characteristics of SnO_2_/rGO sensor in the water molecules adsorption and desorption (11%RH–95%RH–11%RH). (**a**) 1 wt% SnO_2_/rGO, (**b**) 2 wt% SnO_2_/rGO, (**c**) 4 wt% SnO_2_/rGO, and (**d**) 8 wt% SnO_2_/rGO.

Different types of sensitive materials and preparation methods will lead to the different respond speeds of humidity sensors to water molecules during the process of absorption and desorption, resulting in a certain response hysteresis. Typically, the hysteresis characteristic is reflected by the difference in response values during water molecule adsorption and desorption. The sensitive film prepared by electrospinning was transferred from low humidity environment to high humidity environment (11%–20%–34%–56%–77%–95%) and then back to low humidity environment (95%–77%–56%–34%–20%–11%) successively. The red and black curves in Figure 7 represent the change in the capacitance response of SnO_2_/rGO humidity sensor during adsorption and desorption, respectively. Within the whole humidity range, the difference of capacitance values between adsorption and desorption process varies slightly at medium and high RH as shown in Figure 7a–d. Although 1 wt% SnO_2_/rGO sensor has the highest sensitivity, the consistency of its adsorption and desorption curves is the lowest, and the capacitance value varies greatly in high RH. By comparison, the adsorption and desorption curves of other samples (2 wt%, 4 wt% and 8 wt% SnO_2_/rGO) are closer to each other.

**Figure 8:**
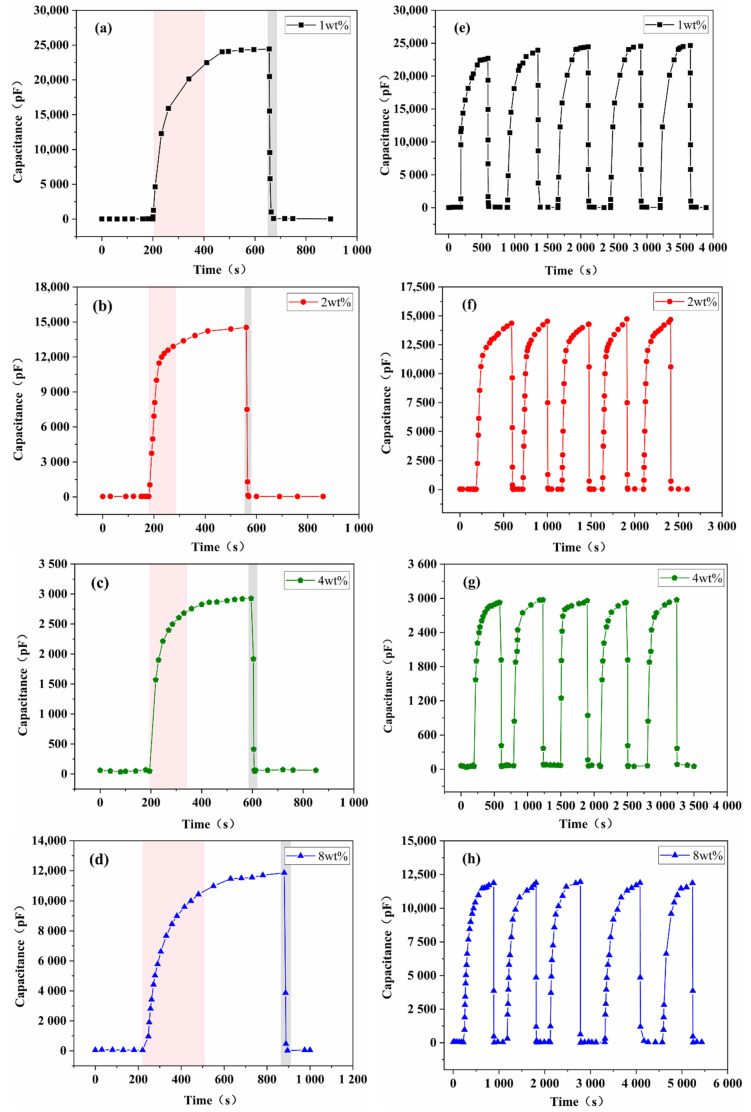
Response/recovery time and repeatability of SnO_2_/rGO sensor exposed to 95%RH from 11%RH with different doping ratios. (**a**,**e**) 1 wt%, (**b**,**f**) 2 wt%, (**c**,**g**) 4 wt%, and (**d**,**h**) 8 wt%.

The testing of the response–recovery characteristics of the sensor was performed. The dynamic response–recovery characteristics curves of the sensors for the different rGO doping ratios in a test circle (11%RH–95%RH–11%RH) are shown in Figure 8. The sensor first reached a stable capacitance value in the 11%RH humidity bottle and remained in it, and then it was quickly transferred to the 95%RH humidity bottle. It can be seen that the capacitance values of all four samples gradually increase and tend to be stable, which indicates that the adsorption of water molecules on the surface of the sensitive film has almost reached saturation at this time. The sensor was then quickly transferred back to the 11%RH humidity bottle. Additionally, it was found that the capacitance value dropped to the initial value in a relatively short period of time. It indicated that most of the water molecules previously adsorbed on the surface of the sensitive film were desorbed. Therefore, the capacitance value of the sensor was basically restored to its original level.

Figure 8a–d shows that the response time (180 s) and recovery time (8 s) of 1 wt% SnO_2_/rGO sensor are approximately twice the response (80 s) and recovery time (4 s) of 2 wt% SnO_2_/rGO sensor. As observed in Figure 8c, although the capacitance response value of the 4 wt% SnO_2_/rGO sensor is the lowest, the response time is not shortened correspondingly and still reaches 120 s. Figure 8d shows that the response time of 8 wt% SnO_2_/rGO sensor is the longest, reaching 280 s. Figure 8e–h shows the repeatability test results of the SnO_2_/rGO humidity sensor. The changes of capacitance response value were recorded by exposing the sensor to 11%RH and 95%RH repeatedly. Under the same condition, the capacitance changes in five repeated cycles are almost negligible, indicating that SnO_2_/rGO humidity sensor exhibits good repeatability and practical value.

### 3.3. Humidity Sensing Mechanism

**Figure 9:**
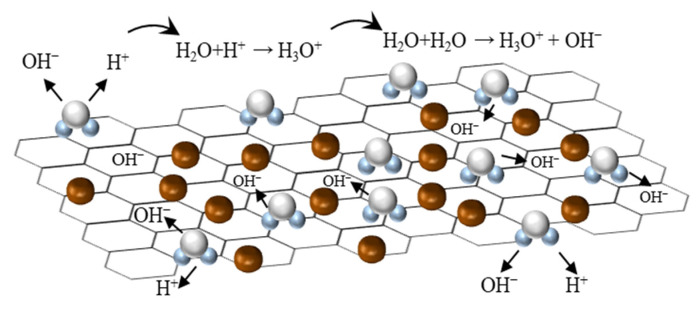
Humidity sensing mechanism diagram of the SnO_2_/rGO sensor.

The process of water molecules adsorption on SnO_2_/rGO film surface was shown in Figure 9. Initially, only a small number of water molecules are attached to the active sites of SnO_2_ and rGO and undergo chemical dissociation to release protons to participate in the conduction process. Since the number of protons is limited and the transfer of protons requires more energy, it results in poor conductivity and small capacitance response. When the adsorption of water molecules starts to increase, more protons are dissociated and combine with other water molecules to form H_3_O^+^ (H_2_O + H^+^→H_3_O^+^). As the relative humidity further increases, lots of water molecules gather to form a continuous water film on the humidity-sensitive film, leading to the enhancement of Grotthuss chain reaction (H_2_O + H_3_O^+^→H_3_O^+^ + H_2_O). This process greatly increases the dielectric constant of the sensitive film, which results in a sharp increase in the capacitance value [41].

The nanostructure of SnO_2_ makes contribution to the rapid transmission of water molecules, which shortens the response/recovery time. Compared to pristine GO, the surface of SnO_2_/rGO nanocomposites possesses a larger specific surface area, which could provide more available active sites for water molecules and accelerate the processes of adsorption and desorption. Thus, the ability of humidity sensing performance is enhanced greatly by the combination of rGO and SnO_2_. It is worth noting that the higher proportion of graphene doping does not mean the better sensing performance. According to the characterization and test results, 2 wt% SnO_2_/rGO shows better humidity sensitivity, which may be attributed to the more uniform distribution of SnO_2_ nanoparticles on rGO layers and the optimal synergetic effect produced by SnO_2_ and rGO.

### 3.4. Bending Test

The flexible humidity sensor is required good conformal feature, which can effectively prevent the cracking of humidity sensitive materials from the substrate and the damage to the sensing structure. Therefore, the bending test of flexible humidity sensor is of great significance to its practical application.

**Figure 10:**
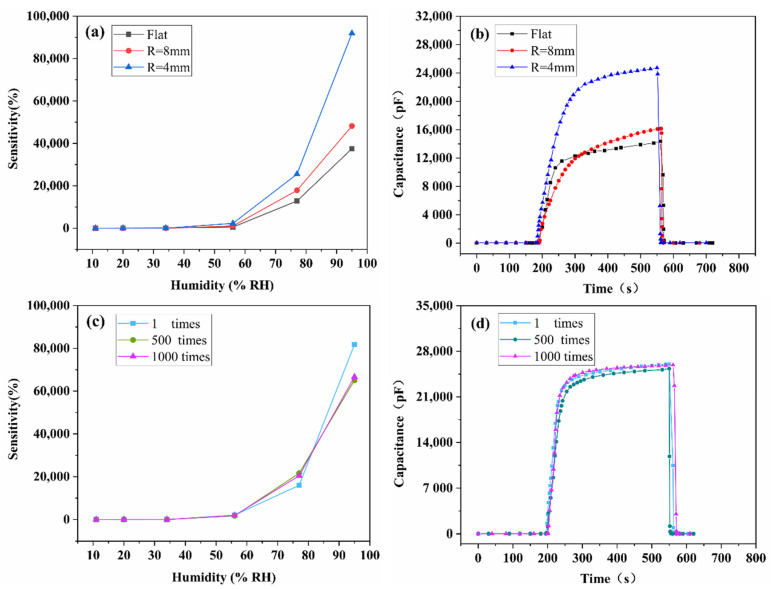
The influence of bending test on humidity performance of 2 wt% SnO_2_/rGO sensor. (**a**) Sensitivity and (**b**) response at different bending radius. (**c**) Sensitivity and (**d**) response after different bending times.

In order to explore the bending effect on the performance of the sensor, the bending test was carried out by attaching 2 wt% SnO_2_/rGO sensor on tubes with different radius. Figure 10a shows that the sensitivity of the sensor at flat and two kinds of bending radius (8 mm and 4 mm) is 37,491%, 48,219% and 91,898%, respectively, indicating that the sensitivity increases as the bending radius decreases. It can be found more clearly from Figure 10b that the capacitance response value of the sensor increases significantly when the bending radius is 4 mm, while the response/recovery time are less affected. The results shows that the contact area of the SnO_2_/rGO film increases during the bending process, which allows the water molecules penetrate more deeply into the sensitive material. The bending radius of 4 mm is chosen as the typical extreme bending condition in practical applications. Therefore, the repeatability test was performed under the condition of a bending radius of 4 mm. Figure 10c shows that the sensitivity after bending 1000 times is slightly lower than that after bending once, while almost the same as that after bending 500 times. As shown in Figure 10d, there is no significant change in response/recover characteristic after bending 1000 times, indicating a good bending property of the SnO_2_/rGO humidity sensor. Bending test results suggest that the prepared sensor has practical potential in wearable devices and applications related to human health.

### 3.5. Monitoring Human Respiration

**Figure 11:**
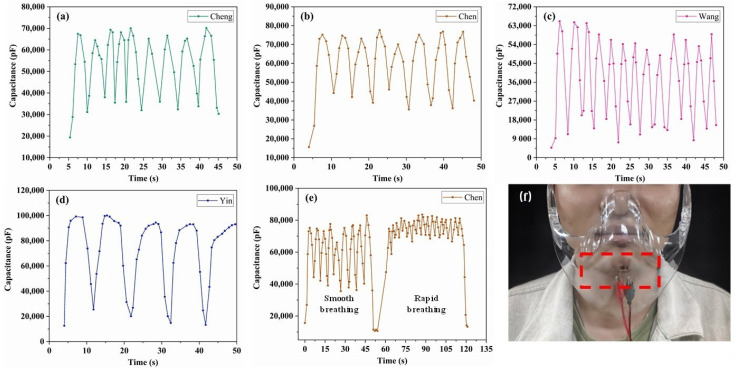
Response curve of human breathing monitoring. (**a**–**d**) Smooth breathing of different adults and (**e**) different breathing states of an adult. (**f**) Sketch map of human breathing test.

Generally speaking, the air humidity exhaled by the human nose and mouth is much higher than 11%RH. That is to say, there is no need to wait for the complete absorption and desorption of water molecules when the humidity sensor is actually applied in breathing monitoring [42]. Therefore, despite the relatively long response/recovery time of the SnO_2_/rGO humidity sensor in 11–95%RH humidity environment, it has a good performance in the human breathing test. The 2 wt% SnO_2_/rGO humidity sensor was selected as the test sensor because of its high sensitivity, fast response/recovery speed. As shown in Figure 11f, the prepared sensor was connected to the inside of a transparent mask and wore on the face of the testee. Due to a small size, the humidity sensor neither obstructed the sight nor affected the wearing experience. The response/recovery characteristics of both different adults and different breathing states were tested, respectively.

Figure 11a–d represented the test curves of four different adults with smooth breathing state. The results show that the sensor has excellent individual selectivity (personalized characteristics), and can accurately reflect the different breathing rates of different testees, which provides a guarantee for its practicality in human respiration monitoring. Furthermore, different breathing states of the same subject were also tested. As shown in Figure 11e, the respiratory interval after exercise is shortened from 5 s to 3 s and the capacitance value is always maintained at a high level (>68,000 pF). It may be caused by the inadequate response/recovery process during rapid exhalation. More importantly, it could be seen that the response signal is not significantly weakened during human breathing tests, indicating the promising application of SnO_2_/rGO humidity sensor for real-time human breathing monitoring.

## 4. Conclusions

In summary, a high-sensitive and flexible humidity sensor based on SnO_2_/rGO nanocomposites was fabricated. The methods of XRD, SEM, TEM and Raman spectroscopy were used to characterize the morphology and composition of the SnO_2_/rGO nanocomposites prepared by hydrothermal method. Electrospinning method is used to spray SnO_2_/rGO sensitive film on PI film for improving the adhesion. More importantly, the nanoparticles could be uniformly dispersed on the film and increase the active sites for water molecules. Humidity test results demonstrated that the SnO_2_/rGO sensor with different doping ratios of rGO show different humidity sensing behaviors. Although 1 wt% SnO_2_/rGO has the highest sensitivity to water molecules, the response/recovery characteristic as well as hysteresis characteristic of 2 wt% SnO_2_/rGO humidity sensor is better. Besides, the sensor exhibits a good response to humidity at different bending radius and the performance remains stable after bending 1000 times. Results of human respiration show that the 2 wt% SnO_2_/rGO humidity sensor could clearly reflect the different breathing states of different adults, making it more suitable for human respiratory monitoring. These results pave a new path to realize SnO_2_/rGO flexible humidity sensor in the application for personal real-time health monitoring as well as other health applications.

## Data Availability

The data presented in this study are available on request from the corresponding author.
